# Indication of Electromagnetic Field Exposure via RBF-SVM Using Time-Series Features of Zebrafish Locomotion

**DOI:** 10.3390/s20174818

**Published:** 2020-08-26

**Authors:** Yaqing He, Kim Fung Tsang, Richard Yuen-Chong Kong, Yuk-Tak Chow

**Affiliations:** 1 Department of Electrical Engineering, City University of Hong Kong, Hong Kong 999077, China; ee330015@cityu.edu.hk (K.F.T.); eeytc@cityu.edu.hk (Y.-T.C.); 2 Department of Chemistry, City University of Hong Kong, 83 Tat Chee Avenue, Hong Kong 999077, China; bhrkong@cityu.edu.hk; 3 Futian-CityU Mangrove Research & Development Centre (FCMC), Futian National Nature Reserve, Mangrove Road, Shenzhen 518040, China

**Keywords:** electromagnetic field (EMF) exposure, ambient environmental parameters (AEPs), zebrafish locomotion, time-series feature, RBF-SVM classification

## Abstract

This paper introduces a novel model based on support vector machine with radial basis function kernel (RBF-SVM) using time-series features of zebrafish (*Danio rerio*) locomotion exposed to different electromagnetic fields (EMFs) to indicate the corresponding EMF exposure. A group of 14 adult zebrafish was randomly divided into two groups, 7 in each group; the fish of each group have the novel tank test under a sham or real magnetic exposure of 6.78 MHz and about 1 A/m. Their locomotion in the tests was videotaped to convert into the x, y coordinate time-series of the trajectories for reforming time-series matrices according to different time-series lengths. The time-series features of zebrafish locomotion were calculated by the comparative time-series analyzing framework highly comparative time-series analysis (HCTSA), and a limited number of the time-series features that were most relevant to the EMF exposure conditions were selected using the minimum redundancy maximum relevance (mRMR) algorithm for RBF-SVM classification training. Before this, ambient environmental parameters (AEPs) had little effect on the locomotion performance of zebrafish processed by the empirical method, which had been quantitatively verified by regression using another group of 14 adult zebrafish. The results have demonstrated that the purposed model is capable of accurately indicating different EMF exposures. All classification accuracies can be 100%, and the classification precision of several classifiers based on specific parameters and feature sets with specific dimensions can reach higher than 95%. The speculative reason for this result is that the specified EMF has affected the zebrafish neural aspect, which is then reflected in their behaviors. The outcomes of this study have provided a new indication model for EMF exposures and provided a reference for the investigation of the impact of EMF exposure.

## 1. Introduction

Human exposure to electromagnetic fields (EMF) has aroused numerous public concerns with the booming development and widespread usage of wireless applications. According to the International Commission on Non-Ionizing Radiation Protection (ICNIRP) standards [1], human exposed to EMF could cause some unfavorable influences on the body. For example, exposure to a low-frequency magnetic field (MF) may induce the electric fields inside the human body, which probably result in adverse human effects by stimulating the nervous system [1,2]. The dosimetry analyzing approaches based on the computational numerical methods using human voxel models or equivalent models have been used to evaluate the potential effects of the EMF on the human body for years. Many outcomes have been carried out and have contributed to the establishment and update of international guidelines [3,4] for protecting humans from the health risk of EMF exposures. 

However, the results of the dosimetry study itself are not sufficient to represent the actual human effects of EMF exposure. The results of practical experimental studies can help assess the impact of EMF exposure on human health more accurately and thus are of great value. However, the actual experimental research also faces considerable challenges [5]. One is that it is difficult to determine the location of the induced current or electric field inside the human body. The other is that directly asking human subjects to investigate the uncertain health hazards caused by EMF may lead to considerable risks. Since ICNIRP recommends and uses animal model evaluation results to consider the potential health risks of electromagnetic radiation to humans [1], are there any alternative instructions or testing tools except for simulation calculations that have been found to represent different levels of EMF exposure?

Biological monitors used in the ecological environment field may provide an alternative solution to this problem. They detect the presence of toxic substances by continuously tracking the physiological performance of organisms [6] and could provide more reliable predictions of substance toxicity than the chemical concentration alone, especially in case of mixtures of substances [7]. The fast development of a fish tracking system supports enhancing the ability of biological monitors—or more specifically, a biological early warning system (BEWS)—to observe and sense the behavioral response of bio-organisms [8,9,10,11,12,13]. The tracking systems of BEWS could provide abundant and explicit information for the behavior and movement analysis, contributing to the satisfying result of water quality assessment when the concentration of pollutants in the water is relatively low. Unlike BEWS, which is committed to detecting harmful substances or water quality defects under the lowest possible concentration conditions, the biological indicator model proposed in this study is used to detect and indicate the specific EMF exposure conditions whose intensity is close to the standard limit. However, similarly, the biological indication model for EMF exposure is expected to have the advantages of BEWS in water quality or toxicity warning, and it needs to satisfy some criteria. Firstly, the animal should have the fundamentals for monitoring the biological aspects of EMF exposure. Secondly, the behavioral responses of the animal should be easily observed, and they can be perceived through recording devices, although high-precision video tracking devices for real-time data evaluation can help read and analyze their performance. Zebrafish (*Danio rerio*) has been most used for this purpose. As a vertebrate species, zebrafish shares as high as 75% genetic homology [14] and substantial physiological homology with mammals [15], and its complex behaviors, including approach –avoidance, cognitive, schooling behavior, and social paradigms [16,17,18,19,20], provide an investigable model to detect disorders from the brain [21] and the nervous system [22]. Therefore, the behavior of zebrafish, such as approach–avoidance, cognitive, schooling behavior, social paradigms, and locomotion, offers a possible model to evaluate any neuron-stimulated impact [16,17,18,19,20]. Among many behaviors, zebrafish locomotion is usually selected [15,20,23,24], and it has been solved as time-series [20] similarly to other animal model studies, e.g., the flying track of the Drosophila melanogaster [25].

On the other hand, some studies have evidenced that zebrafish could sense the geomagnetic field (GMF) or ‘magnetoreception’ [26,27,28], reflecting the impacts of the GMF on the directional actions, embryo, and youth development. The ability of ‘magnetoreception’ may provide the fundamental of effects caused by other types of EMF on zebrafish. Research output has reported the significant impact of the extremely low-frequency EMF and static MF with a higher intensity of the MF strength, e.g., 7.5 mT on zebrafish development [29]. In short, zebrafish have the prerequisites, the foundation, and their own advantages as different EMF exposure indicators. Therefore, it is possible to use zebrafish at varying levels of EMF exposure as a biological indicator of EMF exposure. 

It should be noted that zebrafish are very sensitive to environmental changes and can cause strong reactions, such as jumping out of the tank, etc., which may affect the accuracy of the indication. The empirical method used to solve this problem in other studies is to place the fish in the experimental environment long enough, e.g., 24 h before the experiment. The results of other studies have supported that this method could effectively reduce the stimulation of zebrafish by surrounding environmental factors, but this still needs the verification support of quantitative analysis. The Internet of Things (IoT) system, which has been widely used in many surveillance fields, could help with this verification. The system could record environmental parameters with high precision and minimize human influence.

In this study, environmental stimuli (such as ambient environmental parameters (AEPs), including temperature, humidity, vibration, and light illumination, and a certain intensity of EMF) were considered to affect zebrafish behavior, which could be reflected in zebrafish behavior. Thus, conversely, the environmental stimuli could be indicated by zebrafish behavioral features. Therefore, this study assumes that zebrafish movement performance features can indicate different EMF exposures. So, on the other hand, in this study, it is necessary to exclude the influence of the surrounding environment on zebrafish behavior performance.

This study includes two aspects: (1) using the aforementioned pretreatment method to reduce the effect of AEPs on zebrafish movement and verify its effect; and (2) establishing a new model using zebrafish movement features under different EMF exposure conditions to indicate different EMF exposure conditions. 

In this study, zebrafish behaviors in the novel tank test were collected by video recording, from which the time-series of the zebrafish trajectories were converted. A comparative time-series analysis framework, highly comparative time-series analysis (HCTSA), was used to extract zebrafish motion features, and a minimum redundancy maximum relevance (mRMR) algorithm was used to select the features that are most relevant to environmental stimulus conditions. In the first aspect, regression was performed to verify the influence of environmental parameters on zebrafish novelty exploration behavior. Four types of AEPs were recorded using the integrated IoT system, including temperature, humidity, acceleration, and light intensity. We used mRMR to select zebrafish locomotion features consistent with the AEPs and radial basis function (RBF) kernel-based support vector regression (SVR) to verify the effect of the empirical processing methods, reducing the impact of AEPs on zebrafish behaviors. In the second part, zebrafish locomotion features under different EMF exposure conditions were classified to observe the indication of zebrafish locomotive features on the corresponding EMF exposure. In this session, an MF exposure of 6.78 MHz and about 1 A/m was used with sham or real exposure conditions, and mRMR was used to select the time-series features of zebrafish motion in accordance with the exposure conditions to construct the feature sets. A support vector machine with radial basis function kernel (RBF-SVM) was used for classification to distinguish the time-series characteristics of the zebrafish movement trajectory under different MF exposure conditions.

The rest of the paper is organized as follows. The Materials and Methods section introduces the source of zebrafish, the collection and construction of the zebrafish motion time-series, the calculation and extraction of time-series features, AEP records, MF settings and exposure schemes, classification methods, and experiments program. The Results section shows the verification results of the impact of AEPs on zebrafish behavior and the classification results of the time-series features of zebrafish movement under different MF exposure conditions. The Discussion section follows, and the final section presents the Conclusion.

## 2. Materials and Methods

### 2.1. Zebrafish Acquisition

The AB-strain male zebrafish were used for this study. The fish were cultivated in a 50-L glass tank filled with dechlorinated, charcoal-filtered tap water, of which the salinity was adjusted to 1000 ± 50 μS with Instant Ocean® artificial sea salt, and of which the temperature was maintained at 27 ± 1 °C with a commercial aquarium heater. A top fluorescent tube with a 14:10 light–dark cycle illuminated the water tank used for culturing zebrafish. Fishes were fed with Tetramin® Tropical Flakes and 2-day-old live brine shrimp twice daily. All the fishes used in this study were naïve to experiment. All the animal experiments were conducted following the terms of the License under Animals (control of experiments) Ordinance (Cap. 340) issued by the Department of Health of the Hong Kong Special Administrative Region.

In this study, adult zebrafish were adopted for the two experiments. The physiological state of zebrafish in their adult stage is basically stable, so a small age deviation in the adult stage of zebrafish will not affect the results. The first group of 14 2-year-old adult zebrafish was used to verify whether AEP will affect the zebrafish movement in the new tank test, and the second group of 14 3-year-old zebrafish was used for the classification of different MF exposure.

The fishes were preprocessed using the empirical method to adapt the zebrafish to the environment in which the experiment will be conducted, reducing the impact of environmental factors on their behavior and performance. More than 24 h before the study, we put the fish in the beaker and moved them into the experimental environment, so that they would have enough time to adapt to the experimental situation. The beakers containing the fish were in a 26–27 °C water bath and wrapped in plastic paper printed with vegetation background to prevent fishes from seeing each other and environmental views.

### 2.2. Behavior Test, Video-Tracking, and Time-Series Acquisition

The novel tank tests were used to evaluate the zebrafish behavior, all of which were conducted in the laboratory of the department of chemistry, City University of Hong Kong. In this test, a zebrafish was introduced into an arena to observe its action in a specific range of time, the result of which could provide a general behavior index, especially to a new environment [30]. In this study, the behavior test was performed using the novel tank test. The interior dimension of the acrylic novel tank was 150 mm height × 257 mm long × 70 mm wide, and the height of the water arena adopted in this study was 100 mm. The tank was placed inside a video chamber made of white polyethylene foam (external dimensions of about 210 mm height × 345 mm long × 245 mm wide, and with a thickness of 24 mm) to reduce the visual stimuli from the surrounding environmental views during the test.

In many studies, the duration of the test varies, ranging from 6 min [15,31,32] minutes [33] to as long as 30 min [32]. In this study, the duration of the novel tank test was 15 min, ensuring enough time for the collection of zebrafish behavior. The behavior of the zebrafish during the novel tank test was videotaped for 15 min by a high-resolution camera of 1280 × 720 pixels at a frame rate (FR) of 30 Hz (about 33 ms time step). This sampling rate could ensure the sufficient capture of fast zebrafish movement and provide a reasonable size of the manageable dataset. 

The video-tracking software of EthoVision XT 13 (Noldus Information Technology, The Netherlands) was used to track the locomotive behaviors of novel tank tests and save the trace as Cartesian coordinates. For the preprocessing of the track time-series data, the method of locally weighted scatterplot smoothing was adopted to smooth the collected time-series data and to fulfill the missing points within each dataset of the time-series. 

The time-series dataset of each test named TS contains two sets—x and y coordinates with a period of T minutes, which are represented as follows:

(1) $$TS=\;\left[\begin{array}{ccc} x_{1} & x_{2} & \begin{array}{cc} \cdots & x_{m} \end{array}\\ y_{1} & y_{2} & \begin{array}{cc} \cdots & y_{m} \end{array} \end{array}\right]$$

where m = FR × *T* × 60, representing the total number of the values of each trajectory coordinate time-series.

The time-series can be reorganized into a new time-series matrix according to the specified time length of τ minutes to detect the classification effect of the characteristics of different time-series lengths. A new time length matrix for each zebrafish behavior could be established with the FR of 30 Hz as follows:

(2) $$X\;=\;{\left[\begin{array}{cccc} X_{1} & X_{2} & \cdots & X_{M} \end{array}\right]}^{T}\;=\;\left[\begin{array}{cccc} x_{1} & x_{2} & \ldots & x_{1800\tau }\\ x_{1\;+\;1800\tau } & x_{2\;+\;1800\tau } & \cdots & x_{3600\tau }\\ \vdots & \vdots & \ddots & \vdots \\ x_{1\;+\;\left(M\;-\;1\right)\;\times \;1800\tau } & x_{2\;+\;\left(M\;-\;1\right)\;\times \;1800\tau } & \cdots & x_{1800M\tau } \end{array}\right]$$

where M = 15/τ in this study.

### 2.3. Feature Calculation and Extraction

#### 2.3.1. Feature Calculation and Normalization

In this study, features of the time-series of zebrafish behavior were calculated using the time-series analyzing framework named the highly comparative time-series analysis (HCTSA) [34,35]. For a given time-series, the framework could perform substantial feature extraction using more than 1600 algorithms. More than 7700 features could be computed by the framework, including statistical properties, autocorrelation, power spectra, information-theoretic measures of entropy, and so forth. The abundant feature types could help reveal the substantial difference between different kinds of time-series, such as from genotypes or neural disorders. 

Then, the calculated features were filtered by their qualities. For example, some feature outputs might be null or infinite, which would be abandoned. Normalization was conducted using the Z-score method within each feature set using the following Equation:

(3) $$X\;=\;\frac{\begin{array}{ccc} x\; & -\; & µ \end{array}}{\sigma }$$

where *X* is the resulting value, *x* is the original value, µ is the mean value of the feature set, and σ is the standard deviation of the feature set. Finally, the features of each time-series line could be represented as:

(4) $$F=\;\left[\begin{array}{cccc} F_{1} & F_{2} & \cdots & F_{f} \end{array}\right]$$

where *f* is the total number of the features after filtering and normalization.

#### 2.3.2. Feature Extraction

The features of zebrafish trajectory time-series were extracted using the approach of the mRMR algorism [36,37]. Briefly, the mutual information I of two variables x and y is defined by their joint probabilistic density function  p(x), p(y), and p(x,y):

(5) $$I\left(x,y\right)\;=\;\iint^{\;}p\left(x_{i},y_{j}\right)\log\frac{p\left(x_{i},y_{j}\right)}{p\left(x_{i}\right)p\left(y_{j}\right)}dx\;dy$$

Therefore, the maximum relevance is to find a feature set S with m features {$x_{i}$} that satisfy Equation (6), which approximates the largest dependency with the mean value of all mutual in formulation between individual feature $x_{i}$ and the target class *c*.

(6) $$\;\max D\left(S,c\right),\;D\;=\;\frac{1}{m}\underset{x_{i}\in S}{\sum }I\left(x_{i},c\right)$$

However, it is likely that features selected according to maximum relevance could have rich redundancy; thus, minimizing the redundancy will make the feature set a better representation of the entire dataset. Therefore, the minimal redundancy condition can be added to select mutually exclusive features, which is represented as follows [37]:

(7) $$\min R\left(S\right),\;R=\frac{1}{m^{2}}\underset{x_{i},x_{j}\in S}{\sum }I\left(x_{i},x_{j}\right)$$

Therefore, combining the above two constraints, the algorithm could achieve the purpose of ‘minimal-redundancy–maximal-relevance’ [37], which is defined as:

(8) maxΦ(D,R), Φ = D − R

Using the method could select the features related to the maximum mutual information with their corresponding targeted classes, and of the minimum redundancy, or correlation, with the selected features in the feature sets.

### 2.4. Correlation between Zebrafish Behaviors and the AEPs

#### 2.4.1. Experimental Procedure

**Figure 1:**
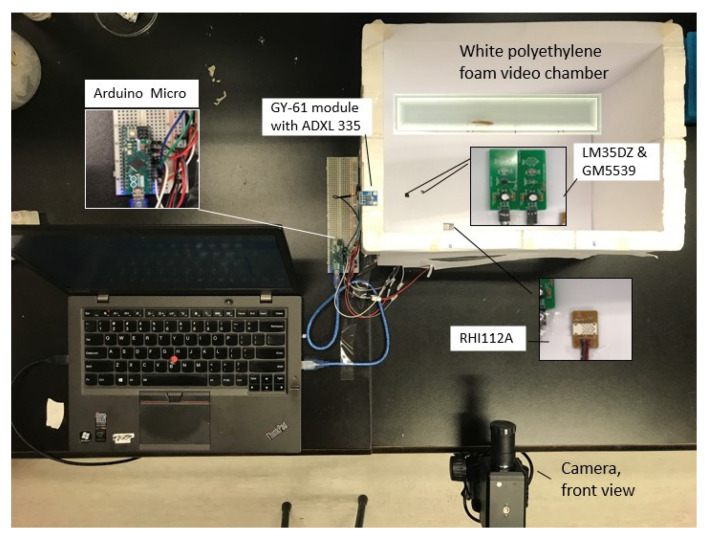
Diagram of the experiment setup for the correlation between the ambient environmental parameters and the zebrafish behavior.

The correlation between the zebrafish locomotion features and the AEPs was firstly conducted to verify the efficiency of the empirical preprocess that reduces the excitation of environment change to zebrafish. In this experiment, a group of 14 2-year-old zebrafish was adopted in this study. After staying in the experimental environment for over 24 h for preprocessing, each zebrafish was removed in the tank filled with reverse osmosis (RO) water to start the behavior test. When the fishes enter into the water arena, the video recording and the AEP recording were started. Each zebrafish had a 15-min novel tank test, after which the videotape and the AEPs recording was stopped synchronously. Then, the fish was moved out of the tank, pouring out the water from the tank and washing the tank with RO water, then adding a certain amount of new RO water to the tank, preparing for the next novel tank test for the next zebrafish. After all of the video records and AEP records were collected, all of the video recordings were converted into the x and y coordinates of trajectory time-series data, and the features of each time-series were calculated using the HCTSA. AEPs were classified for extracting trajectory features using mRMR. Regression was conducted to detect the correlation between zebrafish trajectories and corresponding AEPs. The setup of this study is illustrated in Figure 1.

#### 2.4.2. AEPs Collection

An integrated smart system of an Arduino Micro microchip integrated with four carefully placed sensors was used to record AEPs during the test, as illustrated in Figure 1. The record includes temperature (*P*_T_), humidity (*P*_H_), acceleration in the x, y, and z-axis (*P*_Ax_, *P*_Ay_, and *P*_Az_), and illumination (*P*_I_), with the recording rate of 5 Hz. Here, the acceleration was defined to indicate the vibration of the tank during the novel tank test, which is synthesized by three acceleration values recorded in x, y, and z directions. Specifically, LM35DZ was used to detect temperature; RHI112A was used to record humidity with the percentage output; a GY-61 module with an ADXL 335 accelerometer was used for the vibration or tilt of the video chamber, and GM5539 was used to record the illumination of the video chamber. The sensors were well placed around the tank and covered with white paper to prevent any visual stimulation to the zebrafish. Thus, a piece of raw AEP record was formed as:

(9) $$AEP\;=\;\left[\begin{array}{cccccc} P_{H} & P_{H} & P_{Ax} & P_{Ay} & P_{Az} & P_{I} \end{array}\;\right]$$

The accelerator vector (*P*_A_) was calculated as:

(10) $$P_{A}\;=\;\sqrt{P_{Ax}{}^{2}\;+\;P_{Ay}{}^{2}\;+\;P_{Az}{}^{2}}$$

Thus, each piece of AEP record was formed as:

(11) $$AEP\;=\;\left[\begin{array}{cccc} P_{H} & P_{H} & P_{A} & P_{I} \end{array}\;\right]$$

Thus, we have the matrix of AEP records of *N*-th novel tank test as:

(12) $$P_{N}\;=\;\left[\begin{array}{cccc} P_{T1} & P_{H1} & P_{A1} & P_{I1}\\ P_{T2} & P_{H2} & P_{A2} & P_{I2}\\ \vdots & \vdots & \vdots & \vdots \\ P_{Tn} & P_{Hn} & P_{An} & P_{In} \end{array}\right]$$

where *N* = 1, 2, … , 14, and n is the total number of the records in one novel tank test. Due to the sampling rate (SR) of 5 Hz and the specificities duration T of each novel tank test, the length n of an AEP matrix *P* in each study equals *T* × SR × 60.

#### 2.4.3. Time-Series Establishment for Feature Extraction

To accurately detect the correlation between the AEPs and zebrafish behaviors, time-recording synchronization is essential. In this study, the video recordings and the AEPs used the same laptop, both of which shared the same time keeping from the computer; thus, the two recordings could be synchronized accurately. Furthermore, the corresponding scheme between the AEP and the time-series—more specifically, how to correspond the time-series of trajectories and the AEP records when it is assumed that AEP stimulates the zebrafish during the novel tank test—is another critical issue. In this study, a possible plan for this problem was proposed. An entire time-series was considered to be composed of the numbers of one-second fragments, and for each one-second segment, the AEP at the beginning of the second could be regarded as the environmental stimuli to this segment. Thus, a piece of AEP record corresponded to a one-second segment containing 30 coordinate values of a piece of zebrafish trajectory time-series data. Thus, in this study, the AEP matrices and the trajectory time-series matrices could be reformed as τ = 1 s (1/60 min):

(13) $$TS_{N}\;=\;\left[\begin{array}{cccc} X_{N1,1} & X_{N1,2} & \cdots & X_{N1,30}\\ X_{N2,1} & X_{N2,2} & \cdots & X_{N2,30}\\ \vdots & \vdots & \ddots & \vdots \\ X_{Nl,1} & X_{Nl,2} & \cdots & X_{Nl,30}\\ Y_{N1,1} & Y_{N1,2} & \cdots & Y_{N1,30}\\ Y_{N2,1} & Y_{N2,2} & \cdots & Y_{N2,30}\\ \vdots & \vdots & \ddots & \vdots \\ Y_{Nl,1} & X_{Nl,2} & \cdots & X_{Nl,30} \end{array}\right]$$

and

(14) $$P_{N}\;=\;\left[\begin{array}{cccc} P_{T1} & P_{H1} & P_{A1} & P_{I1}\\ P_{T2} & P_{H2} & P_{A2} & P_{I2}\\ \vdots & \vdots & \vdots & \vdots \\ P_{Tl} & P_{Hl} & P_{Al} & P_{Il} \end{array}\right]$$

where *N* still denotes the *N*-th novel tank test, referring Equation (2), *l* = 15/(1/60) = 900. Then, the HCTSA was used to calculate the feature sets *F_N_* of each *TS_N_* and about 5400 features remained for each time-series after normalization and filtration. The classes of AEP were obtained from the summary of all 14 AEP matrices to ensure that the same feature selection categories were used for each feature set, thus further enabling the inter-datasets correlation exploration. The features from each feature set were selected using mRMR in line with the AEP classes, with each feature set containing 20 feature values that were the most relevant to their corresponding AEP classes. 

Then, *F_N_* was divided into two subsets subject to x and y coordinates, separately, to feature sets *F_x_* containing all selected features from x coordinates, and feature sets *F_y_* providing all selected features from y coordinates, respectively:

(15) $$F_{x}\;=\left[\begin{array}{cccc} F_{1x} & F_{2x} & \cdots & F_{Nx} \end{array}\right]$$

and

(16) $$F_{y}\;=\left[\begin{array}{cccc} F_{1y} & F_{2y} & \cdots & F_{Ny} \end{array}\right]$$

The matrix *P* consisting of all AEP matrices in the sequence was combined as:

(17) $$P\;=\left[\begin{array}{cccc} P_{1} & P_{2} & \cdots & P_{N} \end{array}\right]$$

where *N* = 14. 

#### 2.4.4. Regression

Linear regression was conducted to detect the correlation between zebrafish trajectory features and AEPs during the novel tank test. Support vector machine (SVM) could be used for the regression with a loss function used, and the results have been verified inspiring [38,39]. The basic concept of the SVR is to map the input data x into a higher-dimensional feature space by a non-linear function Φ and perform linear regression in this space. In this study, SVR with a Gaussian kernel function [40] was adopted. 

Then, regressions were conducted between P-containing AEPs and all x coordinate features *F_x_* and between the all APEs P and all x coordinate features *F_y_* with the increasing feature number from 1 to 20 of *F_x_* and *F_y_*. 

Hence, we have:

(18) $$y\;=\;\overset{l}{\underset{i=1}{\sum }}w^{T}\Phi \left(X\right)\;+\;b$$

where y∈ *P*, X ∈ *F_x,_* or *F_y_*, l was the dimension of *F_x_* and *F_y_*; the non-linear function Φ refers to the Gaussian Function. 

The coefficient of determination R^2^ was used, and the root-mean-square error (RMSE) was adopted as the loss function. Thus, in this study, we have

(19) $$R^{2}\;=\;1\;-\frac{\sum_{i=1}^{l}{\left(y_{i}\;-\;y_{i}{}^{’}\right)}^{2}}{\sum_{i=1}^{n}{\left(y_{i}\;-\bar{\;y_{i}}\right)}^{2}}$$

and

(20) $$RMSE\;=\;\sqrt{\frac{\sum_{i=1}^{l}{\left(y_{i}\;-\;y_{i}{}^{’}\right)}^{2}}{l}}$$

where $y_{i}$ and $y_{i}{}^{’}$ denote the measured and predicted values respectively; $\bar{y_{i}}$ refers to the average of $y_{i}$, and n is the number of measurements. These two parameters were used for the evaluation indicators for the model performance.

### 2.5. Zebrafish Behavior under MF Exposure

#### 2.5.1. Experimental Procedure

Then, zebrafish behaviors under MF exposures were collected. In this experiment, the MF with a frequency of 6.78 MHz and an intensity of about 1 A/m was adopted. A group of 14 3-year-old zebrafish was used in this experiment. Seven fishes were under the real exposure, i.e., the aforementioned MF exposure, during the novel tank test; and another 7 fishes were in the sham exposure conditions, i.e., in the same experimental settings but no MF exposure emitted, during their novel tank tests. 

At the beginning of the test, the filming chamber and alignment of the camera was set up. Then, 3 min before filming, the tank was washed and refilled with fresh system water to the certain amount for the test, the outer walls of which were wiped clean. Then, the tank was placed at the designated position inside the filming chamber. Then, the video recording was started soon after the exposure condition: sham or real (randomized). One zebrafish was carefully captured with a certain amount (about half of the volume of the spoon) of water using a black spoon, being held inside the spoon for 5 s; then, it was poured quickly into the novel tank from its central position. The filming lasted for about 20 min, during which we avoided any activity around the chamber. When each test finished, the videotape record was ended at the same time as the test. The water was slowly poured out, and the fish was moved to a bucket for further processing until all the fish used in the experiment were collected. Then, the novel tank was washed and refilled with fresh system water again for the novel tank test of the next fish. The aforementioned procedures of the exposure experiment were repeated until the experiment of the last zebrafish was completed. 

After collection, all of the video recordings were converted into the x and y coordinates of the trajectory time-series data, and they accurately intercepted the time-series data 15 min from the time the zebrafish was placed into the water. Then, the clipped 15-minute time-series were reformed to the time-series matrices by various lengths. Features of each time-series were calculated using the HCTSA and then extracted using the mRMR algorithm. Then, the classification was conducted.

**Figure 2:**
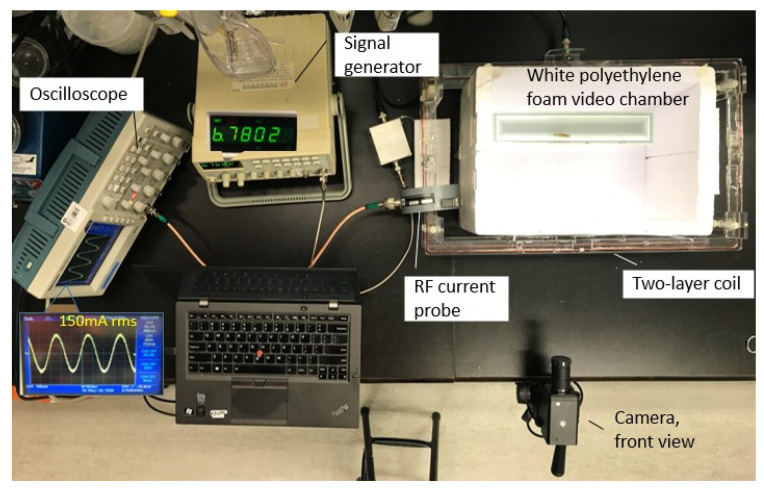
Experimental setting-up of the electromagnetic fields (EMF) on zebrafish behavior during the novel tank test.

Details about the MF are introduced in Section 2.5.1; time-series establishment, feature calculation, and extraction for classification are introduced in Section 2.5.2, and classification is introduced in Section 2.5.3. The setting up of this experiment is illustrated in Figure 2. 

#### 2.5.2. EMF Exposure

**Figure 3:**
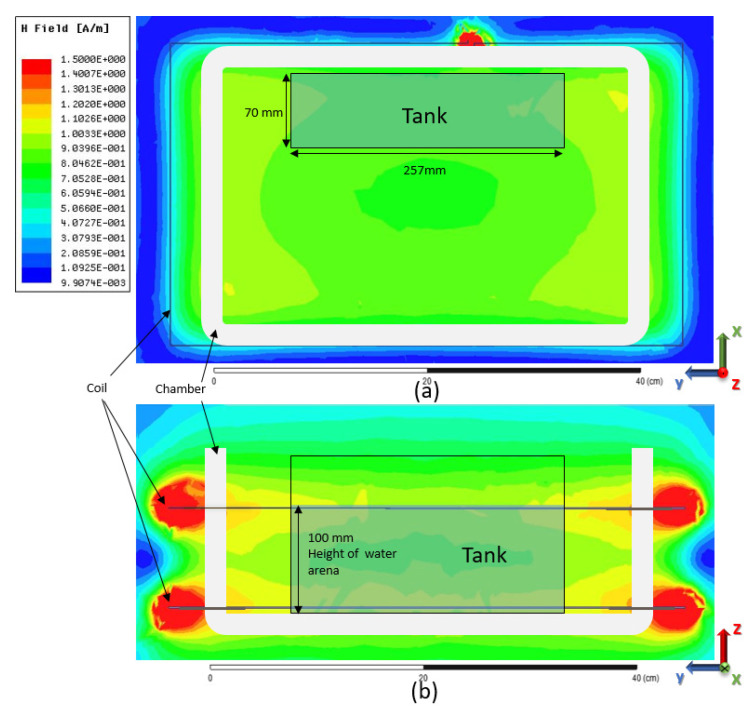
Diagram of the water arena and water tank placed in the induced magnetic field (MF), (**a**) is the top view, while (**b**) is the front view. The location of the MF sectional view in (**a**) is in the middle of the coil of (**b**) on the XY plane, and the site of the MF sectional view in (**b**) is in the middle of the tank in (**a**) on the YZ plane.

The popular frequency of 6.78 MHz, which has been widely adopted for many applications, i.e., wireless charging for the mobile phone [41], wearable devices [42], and electric vehicles [43], was adopted in this study. As Figure 2 illustrates, a 2-layer coil was used to generate a homogeneous MF selected in this study. A newly launched coil [44] that could generate a stable and homogeneous MF inside the coil inside was adopted in this study. The simulation result of the MF distribution of the loop was performed using HFSS 16.1 (ANSYS, Canonsburg, PA, USA), as shown in Figure 3. As Figure 3 illustrates, when the current flowing in the coil was 150 mA root-mean-square (RMS), a homogeneous MF could be induced inside the loop, inside which the intensity where the tank was placed was stably about 1 A/m. The input signal was given by a signal generator (GFG-8210, GW Instek, Taipei, Chinese Taiwan), and the current flow on the coil was monitored by a radio-frequency current flow probe (F42, FCC Inc., Fremont, CA, USA) connected with an oscilloscope (TDS2012b, TEKTRONIX, Beaverton, OR, USA) throughout the whole experiment. During the experiment, the amplitude and the frequency were adjusted in a timely fashion to ensure the current flowing in the coil is 150 m ARMS so that the intensity of the induced MF could be guaranteed.

It is particularly crucial to ensure that the MF of the specified strength entirely wraps the water area. In this study, the height of the coil was 90 mm, while the height of the water area of the novel tank test was 100 mm. Since the dimension of the induced MF would be larger than the size of the coil, as shown in Figure 3, the MF could completely fulfill the actual water area for the novel tank test, ensuring that the zebrafish would be exposed to the MF everywhere in the arena.

Two classes, ‘real’—EMF exposure of 6.78 MHz and about 1 A/m condition and ‘sham’—the fake exposure condition, were used to label the trajectory time-series under the corresponding exposure condition for further feature extraction. Thus, classes for the feature matrices of the time-series of zebrafish trajectories under the different exposure conditions are shown below in Table 1. 

#### 2.5.3. Time-Series Establishment, Feature Calculation, and Extraction for Classification

##### Time-Series Establishment

In this experiment, each time-series of every novel tank test was converted from the whole 15-minute video, meaning that each time-series contains 27,000 coordinate values.

For detecting the classifying effect of features of different lengths of time-series, the time-series were reconstructed by a variable length of τ minutes based on Equation (2). In this experiment, we defined τ = 1, 3, and 5 min to detect how the features of various lengths of time-series could contribute to the classification of EMF exposure. Table 2 below gives a general introduction of the lengths of time-series, the numbers of time-series matrices, and numbers of time-series feature sets for the novel tank test of 14 zebrafish in the experiment.

##### Feature Calculation

All of the time-series was used to extract features by HCTSA. After normalization and filtration, about 5400 features were left for each time-series. Then, the features were extracted using the mRMR method with the labels prescribed in Section 2.5.3 to obtain the feature set *F_N_*. The *F_N_* was divided into 2 subsets, *F_Nx_* and *F_Ny_* for the x and y coordinates separately. Then, *F_Nx_* and *F_Ny_* were used to form feature sets *F_x_* containing all the selected features from the x coordinates, and *F_y_* providing all the selected features from the y coordinates. The feature set F composite features both x and y coordinates, respectively, and it could be represented as:

(21) $$F_{x}\;={\left[\begin{array}{cccc} F_{1x} & F_{2x} & \cdots & F_{Mx} \end{array}\right]}^{T}$$

(22) $$F_{y}\;={\left[\begin{array}{cccc} F_{1y} & F_{2y} & \cdots & F_{My} \end{array}\right]}^{T}$$

and

(23) $$F\;={\left[\begin{array}{cccc} F_{1} & F_{2} & \cdots & F_{M} \end{array}\right]}^{T}$$

##### Feature Extraction for Classification

The mRMR algorithm was used to select features from the results of HCTSA for the classification. According to Equations (6)–(8), in this experiment, *D* represents the maximal relevance between each feature itself and its target class of the sham or real EMF exposure conditions listed in Table 1, and *R* represents the minimal redundancy between itself and other features. Therefore, the features were arranged from high to low according to their own Φ(D−R). The algorithm of mRMR was used to firstly select 20 features from those of 15-minute time-series of two EMFs in this study to reference the performance of the classifier, thereby determining the feature set and feature dimensions required in the study.

**Figure 4:**
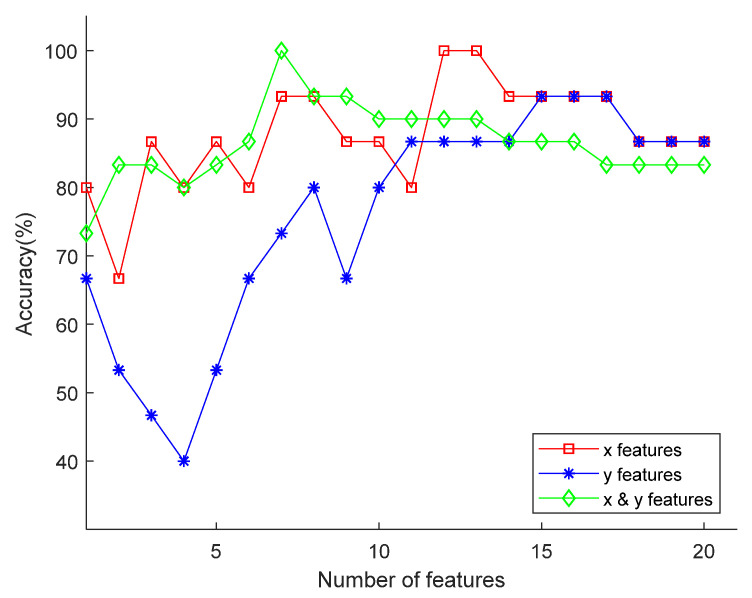
Classification accuracies using feature sets from one-dimensional x coordinates, y coordinates, and x y coordinates composition.

Figure 4 shows the two classification results for two EMF exposure conditions in three dimensions using three feature sets. From Figure 4, for all three feature datasets, within 20 given feature dimensions, the classification accuracy reaches the maximum, and then the classification accuracy decreases due to overfitting. Among them, when a 7-dimensional x and y coordinate combination feature set or a 12 or 13-dimensional x-coordinate feature set was used, the classification accuracy can reach 100%. When a 15-dimensional y-coordinate feature set was used, the classification accuracy can reach 93.3%. The results of the above feature classification accuracy have indicated that the classifier had a more accurate classification effect when using the time-series features of the zebrafish motion x and y coordinates, and the classifier was more efficient. Considering that overfitting occurs due to parameter automation, in this study, the x and y coordinate combination feature matrix was adopted for further progress, and the feature set dimensions (FD) were roughly determined to be 5 and 10. Here, the serial numbers of the 10 extracted features using mRMR for this study are listed in Table 3, and the 5-dimensional feature sets were established with the first 5 dimensions of each 10-dimensional set.

The detailed information of the features corresponding to the serial numbers in Table 3 are elaborated in Table A1 in Appendix A for reference. The serial numbers that are bold in the table illustrate the same features that were selected for the three time-series matrices with various time-series lengths.

#### 2.5.4. RBF-SVM Classification

Support vector machine (SVM), a supervised algorithm of a binary classification solution [38], was adopted in this study. It allows freely choosing model parameters when dealing with high-dimensional and linear inseparable problems, so it has unique advantages such as good generalization ability and robustness [45]. 

Given a training dataset T =$\;{\left\{\left(X,\;y_{i}|X\in R^{m},y_{i}\in \left\{1,-1\right\}\right)\right\}}_{i\;=\;1}^{n}$, where X is an m-dimensional matrix, $y_{i}$ is the binary label, either 1 or –1, and *i* is the feature number

(24) $$\underset{\omega ,b}{argmin}\frac{1}{2}{||\omega ||}^{2}\;,\;s.t.,\;y_{i}\left(\omega^{T}\cdot x_{i}+b\right)\;\geq \;1,\;\forall i$$

where *ω* is a normal hyperplane, and *b* is the offset of the hyperplane from the origin along the normal vector. According to the Lagrange multiplier under the Karush–Kuhn–Tucker condition, (20) could be reformulated as:

(25) $$L\left(\omega ,\;b,\;\alpha \;\right)\;=\;\frac{1}{2}{||\omega ||}^{2}-\overset{n}{\underset{i=1}{\sum }}\alpha_{i}\left(y_{i}\left(\omega^{T}\cdot x_{i}\;+\;b\right)-1\right)$$

where $\alpha_{i}$ refers to the Lagrange multipliers. The partial differential of L(ω, b, α ) with respect to ω results in

(26) $$\omega \;=\;\overset{n}{\underset{i=1}{\sum }}\alpha_{i}y_{i}x_{i}$$

and partial differential of L(ω, b, α ) with respect to b results in

(27) $$\overset{n}{\underset{i=1}{\sum }}\alpha_{i}y_{i}\;=\;0$$

Therefore, a dual Lagrangian problem could be accepted after substituting (22) and (23) into (21)

(28) $$\underset{\alpha }{\arg\;\max}\overset{n}{\underset{i=1}{\sum }}\alpha_{i}\;-\;\frac{1}{2}\overset{n}{\underset{i=1}{\sum }}\alpha_{i}\alpha_{j}y_{i}y_{j}x_{i}^{T}x_{j}\;s.t.\;\alpha_{i}\;\geq \;0,\;\forall i\;\overset{n}{\underset{i=1}{\sum }}\alpha_{i}y_{i}\;=\;0$$

Considering the possible overlap data of two classes that make features non-linear separable, a slack variable ξ and an error penalty constant C were introduced to find a tradeoff between the large margin and the error penalty. Thus, the Lagrangian dual problem was simplified as:

(29) $$\underset{\alpha }{\arg\;\max}\overset{n\;}{\underset{i\;=\;1}{\sum }}\alpha_{i}\;-\;\frac{1}{2}\overset{n}{\underset{i\;=\;1}{\sum }}\alpha_{i}\alpha_{j}y_{i}y_{j}\langle x_{i}^{T}x_{j}\rangle \;s.t.\;C\geq \alpha_{i}\geq 0,\;\forall i\;\overset{n}{\underset{i=1}{\sum }}\alpha_{i}y_{i}=0$$

The Lagrange multipliers $\alpha_{i}$ could be solved by the sequential minimal optimization algorithm [46]. 

The RBF was selected as the kernel function [47] for mapping features into higher-dimensional space that transfer features to be linearly separable in a higher space. For RBF kernel, the Gaussian function was used to map features to a higher dimensional feature space, so that makes the features of the binary classes separable. Thus, we have the $\langle x_{i}^{T}x_{j}\rangle$ in (25), which could be:

(30) $$K\langle x_{i}^{T}x_{j}\rangle =exp\left(-\gamma ||x-x_{i}||{}^{2}\right)$$

where γ (gamma in the context) is the parameter of the RBF kernel that needs to be specified. Thus, we have the classification decision function:

(31) $$f\left(X\right)\;=\;\mathrm{sgn}\left(\overset{n}{\underset{i\;=\;1}{\sum }}\alpha_{i}y_{i}K\langle x_{i}^{T}x_{j}\rangle \;+\;b)\right)$$

where $y_{i}$ is the class label of a support vector, and X refers to the testing feature sets of which the class label is $y_{i}$.

For the each class of the EMF exposure, 5 fishes’ data (71.43%) of each MF exposure condition were randomly selected and used as the training set for training the classifier, and the remaining 2 fishes’ data (28.57%) of each MF exposure were used as the test set for testing the performance of the trained classifier. The penalty parameter C and gamma of the RBF kernel were specified with training the SVM model using different training sets of features. A one-versus-one strategy [48] was selected in this study for the features classification. 

The method of *k* cross-validation [49] was performed for the classification model training in this study to avoid overfitting and to find the best parameter selection. During the training processes, all of the training sets are randomly and evenly divided into k sets, and each dataset has a fixed data ratio of 1:1 between the two classes to avoid training bias. At each fold of validation, k-1 sets are utilized for training the developed model, and the remaining one set is used for validating the trained model, meaning that all data will be validated in the k-fold cross-validation. The dataset used for validation is an “unknown” dataset encountered by the trained model, which renders the evaluation results valid and convincing. After k folds of validation are completed, the classification accuracy can be computed by taking the mean of the accuracies of all the folds. 

In this study, k was decided as 5. Thus, as illustrated in Table 4, for example, when τ = 1 min, there were 420 feature sets of x and y coordinate time-series features for 2 classes. A total of 300 feature sets with an equal number of ‘1’ and ‘−1’ classes were used to train the classifier, and the rest of the remaining 120 sets of two classes were used to test the classifier. When k = 5, the feature set was divided into 5 parts on average, and each part contains an equal number of feature sets labeled as class “1” and “−1”. Four of them were used for model training and the remaining one was used for model testing. This process was repeated 5 times, and the 5 classification rates were averaged to the true classification accuracy of the model.

## 3. Results

**Figure 5:**
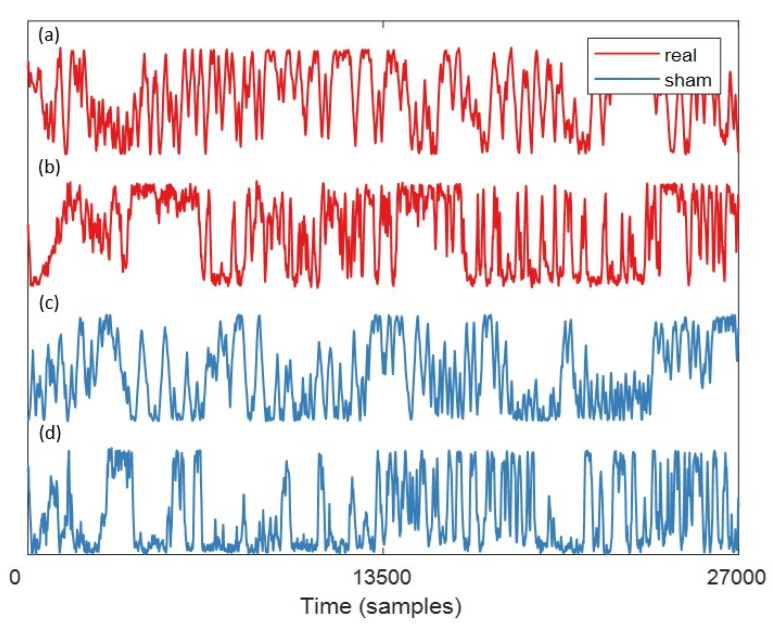
Examples of the trajectory coordinate time-series from tracking their behaviors in the 15-minute novel tank test. The curves show the time-series from the x coordinate of the first zebrafish (**a**), y coordinate of the eighth zebrafish (**b**), x coordinate of the ninth zebrafish (**c**), and y coordinate of the last zebrafish (**d**). Red curves represent the zebrafish under the real EMF exposure condition when having a novel tank test, and the blue curve represents the zebrafish under the sham EMF exposure condition when having the novel tank test.

The time-series of the x and y coordinates of the zebrafish trajectory are shown in Figure 5. At an FR of 30 Hz, each time-series has a total of 27,000 values. In this figure, the minimum and maximum values of the y-axis in Figure 5 represent the arena margins of the novel tank test. Specifically, the minimum and maximum values of (a) and (c) represent the left and right sides of the water area, and the minimum and maximum values of (b) and (d) represent the top and bottom of the water area. It can be observed that zebrafish have more exploration activities from side to side under MF exposure conditions, whether from left to right or top to bottom. On the contrary, when there is no MF, the frequency of such exploration activities was decreased.

Table 5 lists five parameters of zebrafish locomotion under sham and real EMF exposure conditions by each frame time slot: X and Y coordinates of zebrafish, velocity, acceleration, and distance movement. These parameters were adopted for a quantitative description of zebrafish behavior in our study.

From Table 5, it could be seen that the values of five descriptive indexes of adult zebrafish under sham conditions are all higher than those of zebrafish exposed to specified EMF: higher moving velocity and acceleration, further movement distance, and more widespread location in the arena. The result indicated that the zebrafish moved continuously and fast in the absence of EMF exposure, showing strong exploratory activities for the novel environment. On the contrary, the zebrafish under the specified EMF exposure have lower velocity, moving distance, and acceleration, and they are more likely to move around the center of the tank, displaying decreased exploratory activity. The observations from Table 5 are in line with the descriptions from the findings of trajectory coordinate time-series of zebrafish behaviors in the 15-minute novel tank test illustrated in Figure 5.

### 3.1. Verification Result of the AEPs Impact on Zebrafish Locomotion 

The AEPs include temperature, humidity, acceleration, and illustration recorded values surrounding the tank during the novel tank test. Table 6 lists the results of the regression between the trajectory features of the size from 1 to 20 and the individual AEPs.

The results listed in Table 6 indicate that AEPs (including temperature, humidity, acceleration, and light illumination) have almost no linear relationship between the characteristics of the time-series of the x or y coordinates of the zebrafish trajectory. On the one hand, the maximum R^2^ of the regression results derived from the x and y coordinates are 0.03 and 0.04, respectively. They all show that the linear correlation between AEP and zebrafish movement is particularly weak. On the other hand, the negative value of R^2^ in the upper part of the table also indicates that there is no linear correlation between them. The increase of R^2^ in the four items may be due to the rise of the number of features used for regression. In short, there was little correlation between AEP and zebrafish swimming performance during the test. The above results show that after being placed in the test environment for more than 24 h, the behavior of the zebrafish in the new fuel tank test was almost unaffected by the surrounding environment. Quantitative results have shown that the empirical solution is useful for processing zebrafish before the experiment.

### 3.2. Classification Results of the Features of Zebrafish Locomotion

Based on various time-series lengths τ of 1, 3, and 5 min, the size of the corresponding feature set is determined. This operation can also detect how features of time-series in different dimensions contribute to EMF exposure classification. In this study, the time-series features of zebrafish locomotion have been normalized. Therefore, the minimum values of error penalty constant C were set from 2e−10 to 1, and gamma for RBF kernel was selected in the range of [2e−20, 2e20] by classifier training. The feature matrix of the two dimensions (FD = 5 or 10) was used for the classifier training. The following measures have been taken to quantify the effectiveness of the classification system:

(32) $$\mathrm{Accuracy}\;=\;\frac{\mathrm{TP}\;+\;\mathrm{TN}}{\mathrm{TP}\;+\;\mathrm{TN}\;+\;\mathrm{FP}\;+\;\mathrm{FN}}$$

(33) $$\mathrm{Precision}\;=\;\frac{\mathrm{TP}}{\mathrm{TP}\;+\;\mathrm{FP}}$$

(34) $$\mathrm{Recall}\;=\;\frac{\mathrm{TP}}{\mathrm{TP}\;+\;\mathrm{FN}}$$

(35) $$F1\mathrm{Score}\;=\frac{2\;*\;\mathrm{Precision}\;*\;\mathrm{Recall}}{\mathrm{Precision}\;+\;\mathrm{Recall}}$$

where TP, TN, FP, and FN represent the true positive, true negative, false positive, and false negative, respectively [50]. The results of the classification are illustrated in Table 7.

The classification results of SVM with the RBF kernel of the parameters using two dimensions of feature matrices are exhibited in Table 7. From the illustration, the classifying accuracies of the models with different C and gamma using feature sets of the two types of dimensions are 100%. However, the values of precision and recall of the models vary in associated with C, gamma, number of time-series, and the dimension of the feature sets. The best performance of the classifier occurs when the feature set of τ = 1 with 10 dimensions when C = 2e^−10^, of which the precision, recall, and F1 score were 100%, 100%, and 1, respectively. The excellent performances of this feature set also happened in other RBF-SVM models with specified C and gamma values. It was possibly the maximum time-series number and the maximum FD that made this model performance outstanding. The considerable achievement of the classifier occurred when τ = 3 and the dimension of the feature set was 5. When C was increased to 2e^−4^, the values of its precision, recall, and F1-score were 96%, 96%, and 0.96, separately. Then, the performance of the classifier performed better when C continuously increased; the precision, recall, and F1-score increased to 100% (or 1) when C = 2e^−3^, and to 100%, 96%, and 0.9796 when C equals 1. In contrast, when τ = 5, no matter the feature matrix dimension, the precisions of the models were far lower, from 47.619% (Table 7 (e)) to 76.67% (Table 7 (a)). It was possibly because the sample numbers for the time-series matrix of τ =5 were the smallest, which easily led to overfitting. It could be observed that the classifiers would perform better when the dimensions of the feature set were close to the numbers of the time-series sizes. 

When C was too small, overfitting occurred while the RBF-SVMs were trained, which were indicated by the weak values of precisions, recalls, and F1-scores. When C = 2e^−10^ and C = 2e^−5^ (Table 7 (a) and (b)) respectively, the precision values of almost all the models were small, no matter what the value of gamma, and no matter how large the feature set (the feature matrix dimension was either 5 or 10). Larger C values enable better classifier performance. When C = 2e^−4^ and C= 2e^−3^, the performances of RBF-SVM models was increased. There were two models that work better, with the precisions from 96% to 100% and the recalls from 92% to 100% (Table 7 (c) and (d)). When C = 1, the classifiers using feature matrices of τ = 1 and 3 had notable performances; the precision, recall, and F1-score values were larger than 95%, except for the classifying accuracies of 100%.

## 4. Discussion

The purpose of this study is to use the time-series characteristics of zebrafish movement to establish a new model through RBF-SVM to indicate different EMF exposures. To the best of our knowledge, this study is the first to achieve the indication model using zebrafish locomotion trajectory time-series features under different EMF exposures to indicate corresponding types of EMF exposure conditions via the machine learning method. Currently, it is not possible to compare other studies of the same purpose. Differing from BEWS’s propose of detecting low-concentration toxic substances by evaluating the behavior changes of the organisms exposed to it [8,9,12,13], the model of this work is dedicated to indicate the EMF exposure that is near the reference-level field intensity. The behavioral characteristics of zebrafish are selected as the objects for analysis and evaluation, while this study adopted the RBF-SVM model, which is different from existing machine learning models [13] or other statistical models [8,9,10]. Moreover, the features used to train the machine learning model in this study were obtained by the time-series of zebrafish swimming trajectories, which were highly related to EMF exposure conditions. Thus, the experimental results indicated that the RBF-SVM model of this study has achieved higher classification accuracy and prediction precision. Hereafter, the aspects of the reliability of the model, explanation of the findings, and further improvements are discussed

### 4.1. Reliability of the RBF-SVM Models

#### 4.1.1. Classification Accuracy

As demonstrated in Table 7, all of the classifying accuracies of most of the SVM models in this study could be undertaken as high as 100%, which ensures the best classification performances of the SVMs with various parameter identifications. It is because the usage of the mRMR algorithm could ensure that the selected features will enlarge the relevance to the target class, i.e., the classes of different EMF exposure conditions in this study, and minimize the redundancy within the selected feature sets [36]. In this study, HCTSA calculation could provide more than 7000 time-series features, stipulating sufficient feature resources for training the classifier. Then, choosing the appropriate features to improve the performance of the classifier while reducing the impact of noise is crucial to the training of the classifier. From the results, using mRMR to select a limited number of zebrafish motion coordinate time-series features for subsequent classifier training provides an optimized feature scheme for SVM training. On the other hand, the original intention of using mRMR to select features for each time-series matrix is to maximize the relevance between the selected features and the target classification while minimizing the correlation between the selected features. Therefore, the features chosen for different lengths of time-series matrices may be different from the same target class, but there may be some overlap between them. There were three features selected in the time-series features of τ = 1, 3, and 5 min (see Table 3), which are likely to play an essential role in future experiments. 

#### 4.1.2. Robustness

The robustness of this study comes from two aspects. The robustness of this study is guaranteed from the verification of pureeing the effects of AEPs on zebrafish behavior. Zebrafish, the bio-organism used in this study, is an environmentally sensitive species. The difficulty of verifying that zebrafish can be used as an indicator of changes in specific environmental factors is to prove in the experiment that the experimental operation removes the stimulating effects of other environmental factors on it, thereby ensuring the stability and reliability of subsequent research results. In this study, the effectiveness of the AEPs collected by the IoT system on zebrafish behavior has been verified by regression, ensuring that the zebrafish behavior in the novel tank test is only relevant to the EMF exposure. Therefore, in this study, the time-series features of the zebrafish locomotion in novel tank tests are capable of being used to indicate the corresponding EMF exposure via the RBF-SVM classifier.

Besides, the robustness of this study is from the model performances indicated by the values of precision and recall of each model, which is guaranteed by the parameters selection. The changes in the precision rate of the RBF-SVM classifiers were dependent on the different values of the hyper-parameter C and gamma. Generally, the inappropriate choice of error penalty constant C would lead to the SVM model underfitting or overfitting. Therefore, for the data used in this study that has been normalized, if the penalty error penalty constant C is assigned a value of 1 according to the experience of SVM model training, the fault tolerance of the SVM model is too strong to classify accurately. Thus, the strategy of training the model is selecting a minimum C when getting the maximum classifying accuracy. When C was too small, the performance of SVM models with various feature matrix sizes and gamma was unsatisfied. The precisions of the SVM classifier using the feature set were around 54%, separately, when C = 2e^−10^ and C = 2e^−5^. When C of the classifier increases to C = 2e^−4^, the precision increases to 99.33% with an adequate gamma being obtained after training, immediately. For the feature set of τ = 3 and the FD = 5, the precisions were low when C = 2e^−10^ (precision = 75.5102% in Table 7 (a)) and C = 2e^−5^ (precision = 52% in Table 7 (b)). However, the precision of the model increases to 96% when C increases to 2e^−4^, and it increases to 100% when C increases to 2e^−3^. When C gets higher, the ability of tolerance for the SVM classifier will be correspondingly increased, which is also the reason that the precision of each model could obtain 100% when C = 1 and no matter the gamma value. However, the problem is that when the classifiers with different parameters and different dimension feature sets perform well, it means that they all do not perform well. 

#### 4.1.3. Dimension Selections of the Feature Sets

The size of the feature sets used in this study is relevant to the precisions of SVM classifiers. In this study, the time-series matrices of zebrafish locomotion of the novel tank test under the sham or the real specified EMF exposure conditions were re-established with different time lengths and the numbers of time-series of the reformed time-series matrices. For classifiers, a smaller number of feature samples is also more likely to cause overfitting. With the time-series length τ = 5 min, a 15-min time-series of zebrafish locomotion would be reformed to three time lengths. The lower number of time-series resulting in the overfitting happens for all of the SVM models. When using the feature sets with τ = 5, fewer time-series will lead to overfitting for all SVM models, which in turn leads to a decrease in precision during testing. In contrast, the overfitting occurs less when the numbers of the time lengths are equal to or more than the dimensions of the feature matrices, such as feature sets of τ = 1 and τ = 3 min, separately. Therefore, the reasonable quantity of feature samples also has a significant influence on improving the classification accuracy and classification accuracy of the classifier. 

Besides, the dimensions of the feature sets used in this study were referred by the classification accuracy of the full length of zebrafish locomotion time-series of their novel tank tests, of which the maximum dimension was selected as 10. However, the 5-dimensional feature sets of τ = 3 min also made the performance of the classifier considerable with some trained super-parameter C and gamma defined from training. Here, a comparison of the feature dimensions that were processed by the method of principal component analysis (PCA) is conducted. PCA is a method descending from the original FDs that is frequently applied in many studies. Here, PCA was simply applied for the calculated feature sets to reduce their dimensions, as illustrated in Table 8, to be compared with the FDs selected in this study. The thresholds of the cumulative contribution rate of the transformed features were selected as 85% and 95%, respectively, in accordance with the general settings applied in many studies.

Comparing to the numbers of calculated features by HCTSA, PCA could drastically descend the original FDs. However, considering that the size of the feature set selected by the mRMR algorithm used in this study is much smaller than the size of the feature set reduced by PCA listed in the above table, and that excellent performance is obtained in classification, it can be confirmed that the feature selection method used in the study is efficient. On contrast, even if the feature sets with their dimensions reduced by PCA can achieve higher classification accuracies and precisions, the smaller feature set sizes applied in this study could still save a lot of training time and thereby improving the efficiency of the classification model.

### 4.2. Possible Reason behind the Achievement

In this paper, the zebrafish locomotion time-series features under different EMF exposures have been used to indicate the corresponding EMF radiations, and the results have demonstrated a satisfied performance of the models with appropriate parameters. The reference levels of the MF for the general public (or persons in unrestricted environments) and occupational personnel (or persons permitted in restricted environments) prescribed in the international standards/guidelines are summarized in Table 9 [1,3,4,51]. From Table 9, it could be observed that the MF strength used in this study was only slightly lower than that of the reference level value specified for averaging over a 6-min local scheme of occupational exposure in the ICNIRP guideline series, but it was far lower than the strengths prescribed in the IEEE C95.1-2005 and the C95.1-2019. The intensity of the MF used in this study complies with the prescription from the international guidelines. 

Therefore, here, we speculated that the reason for this effect is that the EMF exposure conditions selected in this work have a relatively noticeable impact on the zebrafish nervous system. This effect can be manifested by zebrafish behavior (see Figure 5 and Table 5) and can be quantified by the time-series characteristics of their movement. Here, paired *t*-tests were preliminarily conducted to simply compare the differences of 10 features of the time-series matrices with sham or real EMF exposure conditions of 6.78 MHz and about 1 A/m. The results are exhibited in Table 10. 

From Table 10, it can be observed that the difference among the 10 features of all three time-series matrices under two different EMF exposures is highly significant (*p* <0.001), indicating that the differences in the selected time-series features of the zebrafish locomotion are important for the EMF exposure in this study. This result provides evidence for the apparent difference in the performance of zebrafish under different EMF exposure conditions in this experiment. However, to verify that the electromagnetic radiation exposure conditions given in this study have a significant effect on the neural aspect of zebrafish, other convincing experiments are still required. Some studies have revealed that zebrafish could sense the GMF, or the so-called ‘magnetoreception’, and have reported the impacts of the GMF on the directional actions, embryo, and youth development [26,27,28].

Moreover, the existing international standards, as well as the research agenda, have pointed out that the lower frequency EMF exposure may affect the human neural system [2,4]. Therefore, it could be reasonably inferred that the EMF exposure with a lower frequency and adequate intensity could affect zebrafish’s behavior, which also provide the fundamental of effects caused by other types of EMF on the zebrafish neuron system. The results and the analysis of this study could also provide new support for the investigation of the bio-effects of EMF exposures. In addition to this, is the frequency, or intensity, or the superposition of frequency and intensity of the EMF radiation affecting zebrafish’s behavior? Undoubtedly, more evidence needs to be carried out to validate the impact of the various types of EMF exposures on the zebrafish and reveal the instigating mechanism.

### 4.3. Further Improvement

This study provides a simple and effective model that uses the phenotypic characteristics of the tested animal in terms of movement to indicate different electromagnetic radiation. Beyond this preliminary work, the number of test zebrafish, the number of time series and different lengths, and multi-class classification studies should be considered in the further investigations.

## 5. Conclusions

In this study, novel indication models via RBF-SVM using time-series features of zebrafish locomotion have been achieved for indicating different EMF exposures. The empirical method has been quantitatively verified to be effective in advance to reduce the stimulus of AEPs (temperature, humidity, acceleration, and light illumination) to zebrafish behaviors. 

The locomotion of zebrafish in the novel tank test under sham or real EMF exposure of 6.78 MHz and about 1 A/m intensity was videotaped and then converted into the coordinate’s time-series. The time-series were reorganized into a time-series matrix according to different lengths, in which the characteristics of each time-series were calculated by HCTSA and extracted using the mRMR algorithm under two types of MF exposure conditions. The time-series feature sets of two different dimensions of zebrafish locomotion were used for RBF-SVM training; the hyper-parameter C and the gamma parameter of the RBF kernel were involved in training RBF-SVM within the given range. The results show that using two different dimension feature sets, the RBF-SVM classifiers could obtain high classifying accuracies of 100%; and they have excellent classification performance with precisions higher than 95% with specific parameters of C, gamma, and FDs.

To conclude, the different EMF exposure conditions could be indicated using the time-series features of zebrafish locomotion under corresponding EMF exposures with specific parameters of RBF-SVM classifier and feature set sizes. The reason behind this achievement is inferred that the specified MF exposure has affected the neural aspects of zebrafish and reflected on their behaviors, with the support of the high significances of feature comparisons under sham or real exposures, which needs further investigation.

## Figures and Tables

**Table 1:** Classification of different MF exposure conditions for labeling the time-series feature matrices of zebrafish locomotion.

Class	MF Exposure Conditions
**−1**	Sham MF exposure
**1**	Real MF exposure

**Table 2:** Time-series lengths, numbers of time-series matrices, and numbers of time-series feature sets for the novel tank test of 14 zebrafish.

τ (minutes)	M	Feature Sets	In Total of 14 Zebrafish
1	15	30	420
3	5	10	140
5	3	6	84

**Table 3:** Feature serials extracted using minimum redundancy maximum relevance (mRMR) for time-series matrices of different lengths.

τ	Serial Numbers of Selected Features									
	(1)	(2)	(3)	(4)	(5)	(6)	(7)	(8)	(9)	(10)
1	34	1741	399	15	5209	32	1925	431	1830	3416
3	32	2131	15	36	1902	5366	1712	34	18	2096
5	18	2900	1093	2164	32	36	5400	3774	5401	34

**Table 4:** The numbers of the features sets, numbers of feature sets for training and testing models of two classes, and the numbers of the features with two feature set dimensions (FDs) for each class.

τ (mins)	Feature Sets	Feature Sets for Training	Feature Sets for Testing	Numbers of the Features with Following FDs for Each Class	
				5	10
1	420	300	120	1050	10500
3	140	100	40	350	3500
5	84	60	24	210	2100

**Table 5:** Mean and standard deviation values of zebrafish locomotion under sham and real EMF exposure conditions.

Exposure Condition	Averaged X Coordinate (mm)	Averaged Y Coordinate (mm)	Averaged Velocity (mm/s)	Averaged Acceleration (mm/s^2^)	Averaged Distance Moved (mm)
Sham	−233.25 ± 1.38	−65.41 ± 14.32	50.30 ± 25.15	104.21 ± 52.10	1.68 ± 0.84
Real	52.43 ± 60.95	−36.38 ± 16.65	9.51 ± 14.82	24.42 ± 1.51	0.32 ± 0.49

**Table 6:** R^2^ and root-mean-square error (RMSE) of the regression between overall ambient environmental parameters (APE) summary and up to 20 features from all x coordinate feature matrices, from all y coordinate feature matrices.

Coordinate Time-Series	Feature Dimension	Temperature		Humidity		Acceleration		Illumination	
		R^2^	RMSE	R^2^	RMSE	R^2^	RMSE	R^2^	RMSE
x	1	−0.08	1.0299	−0.02	1.0910	−0.05	1.0854	−0.32	1.1677
	2	−0.09	1.0319	−0.01	1.0843	−0.05	1.0884	−0.24	1.1308
	3	−0.16	1.0660	−0.02	1.0939	−0.09	1.1055	−0.27	1.1478
	4	−0.29	1.1257	−0.06	1.1145	−0.14	1.1301	−0.25	1.1379
	5	−0.27	1.1179	−0.07	1.1209	−0.14	1.1302	−0.32	1.1689
	6	−0.2	1.0825	−0.06	1.1132	−0.06	1.0936	−0.19	1.1102
	7	−0.12	1.0457	−0.03	1.0985	−0.04	1.0801	−0.08	1.0589
	8	−0.07	1.0253	−0.01	1.0850	0	1.0612	−0.03	1.0351
	9	−0.03	1.0033	0.01	1.0777	0.02	1.0498	−0.01	1.0213
	10	−0.01	0.9930	0.01	1.0734	0.03	1.0413	0.01	1.0104
	11	0.01	0.9858	0.02	1.0702	0.03	1.0436	0.03	1.0045
	12	0.02	0.9816	0.02	1.0703	0.04	1.0399	0.02	1.0054
	13	0.02	0.9811	0.03	1.0676	0.04	1.0401	0.03	1.0037
	14	0.02	0.9807	0.03	1.0673	0.04	1.0403	0.03	1.0035
	15	0.02	0.9808	0.03	1.0673	0.04	1.0405	0.03	1.0032
	16	0.02	0.9809	0.03	1.0677	0.04	1.0404	0.03	1.0029
	17	0.02	0.9809	0.03	1.0678	0.04	1.0404	0.03	1.0029
	18	0.02	0.9809	0.03	1.0678	0.04	1.0404	0.03	1.0029
	19	0.02	0.9810	0.03	1.0678	0.04	1.0404	0.03	1.0029
	20	0.02	0.9810	0.03	1.0678	0.04	1.0404	0.03	1.0029
y	1	−0.08	1.0314	−0.02	1.0986	−0.05	1.1027	−0.29	1.1456
	2	−0.11	1.0423	−0.03	1.1038	−0.07	1.1098	−0.2	1.1049
	3	−0.14	1.0579	−0.06	1.1179	−0.06	1.1086	−0.22	1.1118
	4	−0.2	1.0881	−0.05	1.1135	−0.04	1.0945	−0.25	1.1271
	5	−0.18	1.0766	−0.07	1.1226	−0.06	1.1044	−0.2	1.1021
	6	−0.13	1.0542	−0.04	1.1049	−0.04	1.0956	−0.08	1.0486
	7	−0.09	1.0359	−0.02	1.0948	−0.04	1.0951	−0.04	1.0266
	8	−0.06	1.0216	−0.01	1.0904	0.01	1.0700	0	1.0051
	9	0.01	0.9861	0.01	1.0818	0.02	1.0621	0.02	0.9983
	10	0.02	0.9810	0.02	1.0766	0.03	1.0591	0.02	0.9984
	11	0.02	0.9805	0.02	1.0727	0.04	1.0554	0.02	0.9963
	12	0.02	0.9796	0.02	1.0729	0.04	1.0541	0.02	0.9957
	13	0.03	0.9785	0.02	1.0723	0.04	1.0523	0.03	0.9936
	14	0.03	0.9781	0.02	1.0722	0.04	1.0530	0.03	0.9938
	15	0.03	0.9776	0.02	1.0723	0.04	1.0527	0.03	0.9934
	16	0.03	0.9771	0.03	1.0718	0.04	1.0528	0.03	0.9933
	17	0.03	0.9770	0.03	1.0718	0.04	1.0529	0.03	0.9935
	18	0.03	0.9771	0.03	1.0718	0.04	1.0529	0.03	0.9935
	19	0.03	0.9772	0.03	1.0718	0.04	1.0529	0.03	0.9935
	20	0.03	0.9772	0.03	1.0718	0.04	1.0529	0.03	0.9935

**Table 7:** Classification results of different time lengths with optimized parameters with different C_min_ values.

**(a)** **C_min_ = 2e^−10^**							
**τ (mins)**	**Feature Size**	**Parameters**		**Accuracy**	**Precision**	**Recall**	**F1-Score**
		**C**	**Gamma**				
1	5	2e^−10^	0.0039063	100%	54.7619	76.67	0.6389
	10	0.03125	0.0625	100%	100%	100%	1
3	5	2e^−10^	0.03125	100%	75.5102%	74%	0.7475
	10	2e^−10^	0.0039063	100%	70%	56%	0.6222
5	5	1	9.53674e^−7^	100%	76.6667%	76.6667%	0.7667
	10	2e^−10^	9.5367e^−07^	100%	76.6667%	76.6667%	0.7667
**(b) C_min_ = 2e^−5^**							
**τ (mins)**	**Feature Size**	**Parameters**		**Accuracy**	**Precision**	**Recall**	**F1-Score**
		**C**	**Gamma**				
1	5	2e^−5^	0. 0039063	100%	54%	54%	0.54
	10		0.0625	100%	100%	100%	1
3	5		0.03125	100%	52%	52%	0.52
	10		0. 0039063	100%	48%	48%	0.48
5	5		9.5367e^−07^	100%	62.5%	50%	0.5556
	10		9.5367e^−07^	100%	53.33%	53.33%	53.33
**(c) C_min_ = 2e^−4^**							
**τ (mins)**	**Feature Size**	**Parameters**		**Accuracy**	**Precision**	**Recall**	**F1-Score**
		**C**	**Gamma**				
1	5	2e^−4^	0.0039603	100%	74.5902%	60.6667%	0.6691
	10		0.03125	100%	100%	99.3333%	0.9967
3	5		0.03125	100%	96%	96%	0.96
	10		0.0039063	100%	54%	54%	0.54
5	5		9.5367e^−7^	100%	73.33%	73.33%	0.7333
	10		9.5367e^−7^	100%	53.33%	53.33%	0.5333
**(d) C_min_ = 2e^−3^**							
**τ (mins)**	**Feature Size**	**Parameters**		**Accuracy**	**Precision**	**Recall**	**F1-Score**
		**C**	**Gamma**				
1	5	2e^−3^	0.0078125	100%	100%	98.6667%	0.9933
	10		0.03125	100%	100%	100%	1
3	5		0.015625	100%	100%	100%	1
	10		0.0039063	100%	100%	92.5%	0.9583
5	5		9.5367e^−7^	100%	73.3333%	73.3333%	0.7333
	10		9.5367e^−7^	100%	62.5%	50%	0.5556
**(e) C_min_ = 1**							
**τ (mins)**	**Feature Size**	**Parameters**		**Accuracy**	**Precision**	**Recall**	**F1-Score**
		**C**	**Gamma**				
1	5	1	0.00097656	100%	100%	99.3333%	0.9967
	10		0.0039063	100%	100%	100%	1
3	5		0.0019531	100%	100%	96%	0.9796
	10		0.00097656	100%	100%	100%	1
5	5		9.5367e^−7^	100%	66.67%	53.33%	0.5925
	10		9.5367e^−7^	100%	47.619%	66.67%	0.5556

**Table 8:** The numbers of features calculated by highly comparative time-series analysis (HCTSA) and the numbers of features of the time-series matrices after dimension reduction using principal component analysis (PCA).

τ	Total Numbers by HCTSA	Number of Features Getting Cumulative Contribution Rate Threshold	
		85%	95%
1	5210	130	259
3	5367	61	103
5	5401	41	65

**Table 9:** Summary of the MF exposure reference level prescribed in the International Commission on Non-Ionizing Radiation Protection (ICNIRP) and IEEE C95.1 guidelines of 6.78 MHz, *f*_M_ is the frequency in MHz.

Standard/ Guidelines		Schemes	General Public (A/m)		Occupational (A/m)	
			Specification	Value	Specification	Value
ICNIRP	1998	Averaged over 6 min.	0.73/f_M_	0.1077	1.6/f_M_	0.2360
	2020	Averaged over 30 min whole body	2.2/f_M_	0.3560	4.9/f_M_	0.7929
		Averaged over 6 min local	4.9/f_M_	0.7929	10.8/f_M_	1.5929
IEEE C95.1	2005	6 min whole body	16.3/f_M_	2.4041	16.3/f_M_	2.4041
	2019	Averaged 30 min RMS for whole body	16.3/f_M_	2.4041	16.3/f_M_	2.4041
		Averaged 30 min local	36.4/f_M_	5.3687	36.4/f_M_	5.3687

**Table 10:** Comparison results of the features under sham or real specified EMF exposure conditions in each 10-dimensional feature set when τ = 1, 3, and 5 min using paired *t*-test.

τ	Serial Numbers of Selected Features									
1	34 ***	1741 ***	399 ***	15 ***	5209 ***	32 ***	1925 ***	431 ***	1830 ***	3416 ***
3	32 ***	2131 ***	15 ***	36 ***	1902 ***	5366 ***	1712 ***	34 ***	18 ***	2096 ***
5	18 ***	2900 ***	1093 ***	2164 ***	32 ***	36 ***	5400 ***	3774 ***	5401 ***	34 ***

(In the table, *p* < 0.001 (***)).

## Appendix A

**Table A1:** The names and descriptions of the features extracted using mRMR for time-series matrices of different lengths.

Series	Names	Descriptions
**15**	rms	Root-mean-square of the time series
**18**	standard_deviation	Measure of spread of the input time series
**32**	DN_Moments_raw_4	A moment of the distribution of the input time series
**34**	DN_Moments_raw_6	A moment of the distribution of the input time series
**36**	DN_Moments_raw_8	A moment of the distribution of the input time series
**399**	’IN_AutoMutualInfoStats_diff_20_kraskov1_4_ami19’	Statistics on automutual information function for a time series
**431**	’CO_CompareMinAMI_quantiles_2_80_nlocmax’	Variability in first minimum of automutual information
**1093**	’CO_AddNoise_1_std1_10_firstUnder75’	Changes in the automutual information with the addition of noise
**1712**	’CO_Embed2_tau_stdb1’	Statistics of the time-series in a 2-dimensional embedding space
**1741**	’CO_Embed2_Dist_tau_d_ac1’	Analyzes distances in a 2-d embedding space of a time series
**1830**	’NW_VisibilityGraph_norm_olu90’	Visibility graph analysis of a time series
**1902**	’SY_SpreadRandomLocal_100_100_meanac2’	Bootstrap-based stationarity measure
**1925**	’SY_SpreadRandomLocal_200_100_stdac1’	Bootstrap-based stationarity measure
**2096**	’FC_Surprise_dist_50_3_udq_500_uq’	How surprised you would be of the next data point given recent memory
**2131**	’FC_Surprise_T1_100_4_udq_500_median’	How surprised you would be of the next data point given recent memory
**2164**	’FC_Surprise_T2_100_4_udq_500_tstat’	How surprised you would be of the next data point given recent memory
**2900**	’DN_OutlierInclude_n_001_nfexprmse’	How statistics depend on distributional outliers
**3416**	’SC_FluctAnal_2_nothing_50_logi_linfitint’	Implements fluctuation analysis by a variety of methods
**3774**	’NL_crptool_fnn_10_2_ac_fnn7’	Analyzes the false-nearest neighbors statistic
**5209**	’MF_GARCHfit_ar_P1_Q2_engle_mean_diff_p’	Generalized autoregressive conditional heteroscedasticity (GARCH) time-series modeling
**5366**	’ST_MomentCorr_002_02_median_iqr_none_R’	Correlations between simple statistics in local windows of a time series.
**5400**	’MD_rawHRVmeas_SD1’	Heart rate variability (HRV) measures of a time series.
**5401**	’MD_rawHRVmeas_SD2’	Heart rate variability (HRV) measures of a time series.

According to [34], names and descriptions of the first 10 features selected using mRMR for each time series matrix have been listed and introduced in Table A1. Features selected for different time-series matrices relating to the MF exposure conditions were different, but some features were repetitively selected for different time series matrices.
